# How Glyphosate and Its Derivatives Influence Antimicrobial Resistance Emergence and Transmission: A One Health Perspective

**DOI:** 10.3390/antibiotics15040419

**Published:** 2026-04-21

**Authors:** Leticia Malinoski, Gilmar Gonçalves Silva, Larissa Kaniak Ikeda Rodrigues, Leandro Flávio Carneiro, Marcelo Pedrosa Gomes

**Affiliations:** 1 Laboratório de Fisiologia de Plantas sob Estresse, Departamento de Botânica, Setor de Ciências Biológicas, Universidade Federal do Paraná, Avenida Coronel Francisco H. dos Santos, 100, Centro Politécnico Jardim das Américas, Curitiba 81531-980, PR, Brazil; leticia.malinoski@ufpr.br (L.M.); gilmar.silva@ufpr.br (G.G.S.); larissa.kaniak@ufpr.br (L.K.I.R.); 2 Departamento de Fitotecnia e Fitosanidade, Universidade Federal do Paraná, Rua dos Funcionarios, 1540, Curitiba 80035-050, PR, Brazil; leandrocarneiro@ufpr.br

**Keywords:** microbial evolution, horizontal gene transfer, efflux regulation, environmental selection, One Health framework

## Abstract

**Background/Objectives:** Glyphosate-based formulations are globally pervasive pollutants increasingly recognized as potential contributors to antimicrobial resistance (AMR) in environmental microbiomes. Although glyphosate is designed to inhibit plant 5-enolpyruvylshikimate-3-phosphate synthase, it also affects microbial metabolism, stress response, and genetic exchange. This review synthesizes the pathways through which glyphosate, its metabolite aminomethylphosphonic acid (AMPA), and commercial mixtures influence resistance-associated phenotypes and the dissemination of antibiotic resistance (ABR). **Methods:** A critical synthesis of the literature was conducted to evaluate the mechanistic and ecological interactions between glyphosate exposure and bacterial resistance in soil, aquatic, and host-associated microbiomes. **Results:** Experimental evidence showed that sublethal glyphosate exposure induced oxidative stress, altered membrane permeability, activated multidrug efflux pumps, and promoted tolerance phenotypes that could modify antibiotic susceptibility. It also enhances mutation rates and horizontal gene transfer processes associated with the emergence of resistance under controlled conditions. At the community level, glyphosate exposure is associated with microbiome restructuring and enrichment of resistance determinants, often without major shifts in overall diversity of the microbiome. These effects have been reported at environmentally relevant concentrations, although the evidence remains largely derived from laboratory and mesocosm studies. **Conclusions:** Glyphosate acts as both a biochemical modulator of resistance-related phenotypes and an environmental selective pressure that shapes microbial communities. Its widespread use and environmental persistence position it as a context-dependent contributor to the emergence and dissemination of AMR through interacting mechanistic and ecological pathways. Integrating AMR endpoints into pesticide risk assessments and surveillance frameworks is warranted, in addition to expanded field-based validation.

## 1. Introduction

Glyphosate (N-phosphonomethyl glycine) is the most widely used herbicide globally, with hundreds of thousands of tons applied annually in agricultural and urban settings [1,2]. Its popularity stems from its broad-spectrum efficacy against weeds via the inhibition of 5-enolpyruvylshikimate-3-phosphate synthase (EPSPS), an enzyme in the shikimate pathway that is involved in the synthesis of aromatic amino acids in plants [2]. However, the EPSPS pathway is also present in bacteria and some fungi, indicating that glyphosate has inherent antimicrobial properties [1,3]. Glyphosate was originally patented as an antibiotic and metal chelator, reflecting its ability to bind divalent cations (e.g., Zn, Cu, and Mn) and disrupt microbial metabolism [1,2]. Its broad environmental dissemination has raised concerns regarding its non-target effects on microorganisms that also rely on the shikimate pathway for aromatic amino acid biosynthesis. Its persistence and mobility in soils, surface waters, sediments, and food webs ensure chronic exposure of microbial communities across diverse ecosystems, frequently in combination with additional stressors such as metals and pharmaceuticals [4,5]. Despite increasing regulatory pressure and partial bans in parts of Europe and North America, glyphosate continues to be widely used across the Global South, and its environmental persistence ensures prolonged exposure, even when its application is declining in some regions.

Over the last decade, mounting evidence has suggested that sublethal exposure to glyphosate may promote antimicrobial resistance (AMR) or tolerance in bacteria [3,6,7]. This phenomenon, sometimes termed “cross-resistance” or “co-selection,” has raised concerns that heavy herbicide use in agriculture could be an overlooked contributor to AMR dynamics in environmental microbiomes and in pathogens. Unlike direct antibiotic selection, glyphosate’s influence is both direct and indirect, involving biochemical effects on microbial physiology and broader ecological interactions. Glyphosate can alter microbial community composition (dysbiosis) and induce stress responses that may increase mutation rates and horizontal gene transfer (HGT) under specific conditions [8,9]. In addition, experimental evidence indicates that glyphosate exposure can directly modulate antibiotic susceptibility through mechanisms such as efflux pump activation, changes in membrane permeability, and stress response regulation [6]. These effects span multiple environments, including soil and rhizosphere microbiota repeatedly exposed to herbicide runoff, aquatic communities contaminated with glyphosate-based herbicides, and the gut microbiota of animals that ingest glyphosate residues. They are often observed at environmentally realistic concentrations and under chronic exposure regimes that are characteristic of intensively managed agricultural landscapes [3,6,7], with reported environmental levels ranging from low μg/L concentrations in surface waters to mg/kg concentrations in agricultural soils, and substantially higher transient peaks documented in runoff-impacted systems [4,5,10,11]. However, most supporting evidence is derived from controlled laboratory or mesocosm studies, and extrapolation to natural systems remains limited. Under the One Health paradigm, these environmental resistome changes are pertinent to public health because bacteria (and their resistance genes) move between the environmental, animal, and human compartments of ecosystems. Recent findings of clinically significant resistant bacteria in wildlife and natural waters [12,13,14] underscore the fact that environmental reservoirs of AMR can ultimately affect human health.

The central concern of this review is whether the pervasive presence of glyphosate, along with its primary metabolite aminomethylphosphonic acid (AMPA) and commercial formulations, can contribute to the evolution and dissemination of AMR in environmental bacteria through direct and indirect interactions that are context-dependent across environmental conditions. We synthesized mechanistic, ecological, and evolutionary evidence linking glyphosate exposure to shifts in resistomes, emphasizing the potential pathways by which herbicide use may influence AMR emergence and transmission within the One Health framework in the future. Current reviews either focus narrowly on toxicology or treat AMR as a peripheral outcome, leaving gaps in the mechanistic synthesis and environmental integration of AMR. This review addresses this gap by consolidating recent evidence on how glyphosate and its derivatives shape AMR evolution through physiological perturbations, ecological disturbances, and gene flow across environmental matrices and host microbiomes. We argue that understanding herbicide–AMR interactions is timely and necessary to anticipate legacy effects, guide surveillance, and inform policy under a One Health paradigm, as the ecological and public health consequences of glyphosate use are likely to persist beyond its regulatory life span.

## 2. Mechanistic Pathways Linking Glyphosate to AMR

### 2.1. EPSPS Inhibition, Nutrient Starvation and the Stringent Response

Glyphosate’s primary mode of action, the inhibition of EPSPS in the shikimate pathway, can impose metabolic stress on microbes by depriving them of essential aromatic amino acids (phenylalanine, tyrosine, and tryptophan). In susceptible bacteria (particularly those with the Class I EPSPS enzyme, such as many Proteobacteria), glyphosate exposure mimics amino acid starvation [1]. Recent studies have shown that bacteria respond to glyphosate-induced amino acid limitation by activating the stringent response, an evolutionarily conserved stress pathway mediated by alarmone nucleotides (p)ppGpp [15]. Elevated (p)ppGpp levels signal cells to slow down growth and redirect resources, leading to a transiently tolerant state in the cells. Ospino and Spira [15] demonstrated that *Escherichia coli* exposed to glyphosate accumulated (p)ppGpp via RelA (an indicator of the stringent response) and entered a quasi-dormant state, thereby increasing tolerance and persistence to multiple antibiotics without changing the intrinsic minimum inhibitory concentration (MIC). In their study, glyphosate pre-treatment did not increase the MIC for ampicillin, ciprofloxacin, or kanamycin (i.e., no stable resistance mutation was conferred); however, it significantly enhanced bacterial survival during antibiotic exposure by increasing the fraction of persistent cells [15]. This finding suggests that glyphosate can provide a transient survival advantage during antibiotic exposure by promoting persistent phenotypes. While such tolerance is reversible (not genetic resistance per se), it poses clinical concerns by allowing bacteria to survive longer courses of antibiotics, potentially facilitating the eventual emergence of truly resistant mutants.

Nutrient stress from EPSPS blockade may also select for mutant strains with altered metabolic pathways or glyphosate-insensitive EPSPS. Many bacteria can acquire tolerance to glyphosate toxicity via mutations in EPSPS target or regulatory genes that mitigate the effects of herbicides. Liao et al. [6] observed positive selection in soil microcosms for genotypes carrying mutations in known herbicide target genes and antibiotic resistance genes (ARGs) under chronic glyphosate exposure. This implies that some mutations may concurrently confer glyphosate tolerance and antibiotic resistance, for example, through pleiotropic effects on cell physiology or via gene linkage to mobile genetic elements. Although the molecular details remain underexplored, one hypothesis is that bacteria with innate or acquired glyphosate insensitivity (e.g., via a Class II EPSPS enzyme variant) might gain a competitive advantage in herbicide-treated environments, and if these strains carry ARGs, the ARGs are enriched [3]. In this context, glyphosate can act as both a selective environmental filter and a biochemical stressor capable of modulating resistance-associated phenotypes.

### 2.2. Microbial Dysbiosis and Community Shifts

Beyond individual cells, glyphosate can drive dysbiosis in complex microbial communities, potentially favoring opportunistic and resistant taxa in the gut microbiome [8]. Glyphosate differentially affects microbes depending on their reliance on the shikimate pathway or ability to metabolize glyphosate; therefore, even low-level exposures can alter community structure [16]. In soils and plant rhizospheres, glyphosate application has been shown to subtly shift bacterial composition; for example, some beneficial symbionts and sensitive decompositional bacteria decline, while glyphosate-tolerant strains (including certain pathogens or spore-formers) increase [6,8,17]. Yang et al. [18] reported that repeated herbicide applications enriched soil ARGs and mobile genetic elements (MGEs) without major changes in total bacterial diversity or biomass, suggesting that resistome restructuring may occur independently of large taxonomic shifts. These observations indicate that glyphosate exposure is linked to ecological selection processes that can interact with, rather than replace, the direct mechanistic pathways influencing resistance phenotypes.

Glyphosate-induced dysbiosis has also been documented in the host-associated microbiomes of animals [19,20]. Glyphosate residues in feed or water can disturb the gut microbiota of animals [20]—an emerging One Health concern given the microbiome’s role in health and disease. In zebrafish, glyphosate exposure leads to gut dysbiosis and affects the microbiome-gut-brain axis, altering neurotransmitter levels and behavior [21]. Rat studies have demonstrated that glyphosate-induced gut microbiota dysbiosis contributes to male reproductive toxicity through increased IL-17A production [22]. A recent study on wild deer found significant alterations in the gut bacterial community and resistome in animals with higher glyphosate exposure [23]. Although confounded by co-exposure to mycotoxins, deer with greater herbicide residue had distinct microbial profiles and increased abundance of ARGs in their intestinal metagenome [23]. This suggests that dietary glyphosate may select for resistant gut microbes or mobile resistance elements, even in wild herbivores, raising the possibility of wildlife becoming reservoirs or conduits for resistance genes in the food chain. Similarly, earthworms exposed to glyphosate-based herbicides exhibit gut dysbiosis and an elevated abundance of resistance determinants [24]. In that pilot study, three species of soil earthworms showed significant shifts in their intestinal microbiota after chronic Roundup exposure, including the enrichment of bacteria carrying human-pathogenic virulence factors and ARGs [24]. These results highlight that non-target organisms can develop microbiome perturbations under herbicide stress, potentially impacting their health and the resistome of their associated environments.

Even pollinators and insects show glyphosate-driven dysbiosis; for instance, honeybees fed with trace amounts of glyphosate suffer loss of key gut *Snodgrassella* and *Gilliamella* symbionts and become more susceptible to opportunistic infections [25]. Although that study did not measure resistance genes, it demonstrated how glyphosate can undermine normal microbial balance and immunity, possibly creating niches for more hardy, potentially antibiotic-resistant microbes to colonize the host gut microbiome. In humans, direct evidence is limited; however, bioinformatics predictions suggest that approximately 50% of gut bacterial species are inherently sensitive to glyphosate [26]. Some in vivo rodent studies have reported gut dysbiosis and functional perturbations after chronic low-dose glyphosate or Roundup exposure [20,22], although the translation of these findings to human health remains controversial. In mice, glyphosate at doses approximating the U.S. The Acceptable Daily Intake (approximately 1.75 mg/kg body weight/day) significantly impacts the gut microbiota and increases proinflammatory responses [20]. Overall, the community-level effects of glyphosate, although subtle, may shift competitive dynamics toward taxa that tolerate chemical stress and exploit altered niches. Importantly, these ecological dynamics may reinforce resistance-related traits through interactions with cellular stress responses and genetic exchange mechanisms, as described in the subsequent sections. These outcomes are context-dependent and influenced by environmental conditions, microbial composition, and co-occurring stressors; therefore, they should not be generalized across ecosystems.

### 2.3. Induction of Efflux Pumps and Other Stress Responses

Bacteria exposed to sublethal toxins often activate broad stress-response regulons. Glyphosate is no exception: it can trigger pathways associated with xenobiotic detoxification and membrane protection. One key mechanism is the upregulation of efflux pumps, which are multidrug transporters that expel toxic compounds from cells [27]. Efflux pumps are a common mechanism of antibiotic resistance (especially for multiple drug classes), and evidence suggests that glyphosate exposure can select for or induce efflux pump activity [7]. Bacterial communities chronically exposed to glyphosate are enriched in multidrug efflux pump genes, presumably because these pumps confer a fitness advantage in surviving glyphosate toxicity [7]. This implies that efflux systems that are effective against herbicides may also pump out antibiotics, linking glyphosate tolerance to cross-resistance. Mechanistically, glyphosate is a polar anion that may not diffuse freely across the cell membrane [28]. Bacteria that increase efflux or reduce permeability can limit intracellular glyphosate accumulation, thereby reducing the antibiotic influx. Liao et al. [6] observed that soil bacteria under herbicide selection pressure acquired mutations not only in target genes but also in regulatory regions that could boost efflux pump expression, supporting this idea.

Molecular studies have further supported this association. For example, Kurenbach et al. [29] reported that *E. coli* and *Salmonella* exposed to glyphosate showed increased MICs to antibiotics like ciprofloxacin and kanamycin, and transcriptomic analysis pointed to activation of the AcrAB-TolC efflux system and other stress regulons during herbicide exposure. Similarly, Liao et al. [6] demonstrated that glyphosate and glufosinate exposure in soil microcosms significantly increased the frequency of multidrug resistance plasmid transfer, a phenomenon partly attributed to higher cell membrane permeability (discussed below) and possibly to the stimulation of efflux-regulated conjugation genes. Additionally, *Pseudomonas aeruginosa* isolates pre-exposed to glyphosate-based herbicides showed overexpression of MexAB-OprM efflux pumps and downregulation of the OprD porin, correlating with decreased susceptibility to imipenem [30]. Efflux overexpression and porin loss are classic mechanisms of carbapenem resistance in *P. aeruginosa*. Háhn et al. [28] found that sublethal concentrations of glyphosate-based herbicides could induce a 2–31-fold increase in imipenem MIC in some strains. These findings demonstrate that glyphosate exposure can directly alter antibiotic susceptibility profiles through mechanisms such as efflux activation and membrane remodeling. Although the exact regulatory triggers remain under study, this suggests that glyphosate formulations can phenotypically “train” bacteria to activate resistance nodules (such as efflux pumps) that span multiple chemical classes. However, these responses are highly strain-specific and depend on exposure conditions, limiting their direct extrapolation to environmental systems.

In addition to efflux, glyphosate stress likely engages the general stress response (controlled by RpoS in many bacteria) and other defense mechanisms [31,32]. RpoS induction could contribute to an overall resilient state with lower membrane permeability and higher expression of detoxification enzymes, which, in turn, reduces the efficacy of antibiotics [33]. There is also evidence that glyphosate exposure generates reactive oxygen species (ROS) in non-target microbes [34,35], leading to oxidative stress. ROS can damage cellular components, prompting bacterial antioxidative defenses and SOS DNA repair pathways (discussed in the following section). Freitas et al. [33] observed that a soil *Pseudomonas fluorescens* activated specific quorum-sensing molecules and altered its membrane fatty acid composition in response to glyphosate, likely as an adaptive mechanism to mitigate oxidative membrane damage. These changes are associated with enhanced tolerance to herbicides and environmental stressors. Collectively, the induction of intrinsic resistance mechanisms (such as efflux and membrane modifications) upon glyphosate exposure underscores a trade-off: bacteria invest energy in broad protective functions that incidentally confer cross-protection against antibiotics [36]. This phenomenon aligns with the concept of “stress-induced cross-tolerance,” wherein surviving one stress (herbicides) equips microbes to better survive another stress (antibiotics). However, it is important to note that these adaptations may carry fitness costs in the absence of stress, which is an area for further research on the stability of glyphosate-selected traits in natural populations. These responses may confer cross-protection and directly modify antibiotic susceptibility profiles under certain conditions.

### 2.4. Oxidative Stress, DNA Damage and Mutation

Sublethal herbicide exposure creates a biochemical environment conducive to genetic changes. Glyphosate, especially certain surfactants in formulations (such as polyethoxylated tallow amine, POEA), can cause membrane perturbations that lead to excess ROS inside bacterial cells [34,35]. Elevated ROS levels can damage DNA, and bacteria respond by activating the SOS response, a global pathway controlled by RecA/LexA that induces DNA repair and error-prone polymerase activities [37,38]. Activation of the SOS response is known to enhance mutation rates and facilitate HGT (through integrase and transposase activation), processes that are closely associated with antibiotic resistance evolution [37,39]. Although direct evidence of glyphosate-triggered SOS in pathogens is still emerging, parallel examples exist for other redox-active pollutants (e.g., quinones in tire leachate) that induce SOS and increase antibiotic resistance gene mobilization [40]. Similarly, glyphosate-induced oxidative stress may subtly promote mutagenesis and horizontal gene transfer. Experimental evidence supports this relationship. One study reported that *E. coli* exposed to low concentrations of glyphosate (0.0067 g/L) had an increased frequency of rifampicin-resistant mutants (six times compared to spontaneous levels), possibly linked to transient hypermutation during stress [41]. These findings provide experimental evidence that glyphosate-induced stress can increase mutation frequency and the likelihood of resistance emergence under controlled conditions, although evidence of consistent effects across environmental systems remains limited. Overall, oxidative stress and DNA damage should be interpreted as contributors to evolutionary processes, particularly under sublethal exposure scenarios in which adaptive responses are triggered. There is also a link between glyphosate exposure and specific resistance mutations in the target genes. Raoult et al. [3] noted that *E. coli* and *Salmonella enterica* cultures grown with glyphosate became less susceptible to quinolone antibiotics, in part due to mutations in gyrase or topoisomerase genes (the targets of quinolones). They posited that glyphosate-induced cellular stress might select for mutants with altered target sites or global regulators that incidentally reduce antibiotic susceptibility [3]. Additionally, glyphosate interference in DNA replication (through nucleotide pool imbalances or ROS damage) can pause replication forks and trigger LexA cleavage (SOS induction), which upregulates integrases and recombinases that mobilize gene cassettes and plasmids. For example, *E. coli* exposed to oxidative stress often show increased rates of plasmid recombination and transfer [42]. Recent evidence indicates that glyphosate can fit this pattern: at environmentally relevant concentrations (10–20 mg/L), glyphosate significantly boosted the conjugative transfer rate of a multi-antibiotic-resistant plasmid in laboratory mating experiments [43]. The authors found that glyphosate-treated donor and recipient bacteria had higher membrane permeability and likely upregulation of mating pair formation genes, facilitating plasmid uptake by recipient bacteria [43]. Although the exact molecular triggers (e.g., SOS vs. other stress regulons) have not been fully delineated, the outcome is clear: glyphosate can increase HGT between bacteria, spreading ARGs even in the absence of antibiotics.

Another aspect to consider is whether the chemical structure of glyphosate directly causes DNA damage or induces replication errors. Glyphosate is not a classic DNA-intercalating mutagen; however, chronic exposure may lead to indirect DNA damage via ROS production or misincorporation of analogs [43,44]. There has been speculation (though not conclusively proven) that glyphosate, being an amino acid analog of glycine, could be mistakenly incorporated into peptides or interfere with protein synthesis fidelity, potentially leading to proteotoxic stress and further oxidative damage to cells [45]. These hypotheses remain underexplored; however, any process that increases the mutation supply in a bacterial population (even slightly) can accelerate the emergence of antibiotic-resistant bacteria. Thus, glyphosate-induced oxidative and DNA stress responses can be viewed as catalysts for microevolution, potentially accelerating the rate at which bacteria adapt to antibiotics or acquire new resistance mechanisms against them. Importantly, such mutagenic or recombinogenic effects strengthen the case that environmental chemicals, such as herbicides, traditionally assessed only for acute toxicity, might also exert “evolutionary toxicity” by increasing the genetic variability on which natural selection for antibiotic resistance can act.

### 2.5. Horizontal Gene Transfer Facilitation

Perhaps the most striking mechanism linking glyphosate to AMR is the facilitation of HGT in bacteria. Even if glyphosate does not cause a new resistance mutation, it can aid in the spread of existing ARGs. Recent studies in both soil and water contexts have demonstrated that glyphosate exposure can escalate the exchange of plasmids, transposons, and other mobile genetic elements (MGEs) that carry ARGs in bacteria. Liao et al. [6] found that soil bacteria exposed to glyphosate (and other herbicides) showed a higher abundance of integron-integrase genes and plasmid-associated genes, indicating HGT events. They directly measured that herbicide treatment increased the conjugation frequency of a multi-drug resistance plasmid by up to an order of magnitude compared to that of unexposed controls [6]. Mechanistically, glyphosate appears to facilitate bacterial DNA sharing by (i) increasing cell membrane permeability and (ii) stimulating cell-to-cell contact or competence. Glyphosate causes subtle membrane depolarization and permeability changes in *Pseudomonas fluorescens* [46], which can facilitate mating bridge formation during conjugation. Essentially, glyphosate may put bacteria into a “sharing mode,” possibly as a survival strategy. When stressed by a non-lethal toxin, microbes may benefit from acquiring new genetic material (which is analogous to how antibiotics at sublethal doses induce SOS and HGT). This demonstrates a direct mechanistic pathway through which glyphosate exposure enhances the dissemination of antibiotic resistance genes. However, these effects have predominantly been demonstrated in controlled systems, and their magnitude and persistence in natural environments remain uncertain. Therefore, glyphosate should be considered a modulator of gene transfer dynamics with experimentally demonstrated effects, although its impact across heterogeneous environmental conditions requires further investigation.

In addition to conjugation, other HGT routes, such as transformation (uptake of free DNA) and transduction (phage-mediated transfer), can influence HGT. Environmental stress often induces transformation competence in some species (e.g., *Acinetobacter* and *Bacillus*) [47,48]. In aquatic microbiomes, Barbosa da Costa et al. [49] provided field-based evidence of cross-selection: in freshwater mesocosms, Roundup treatment led to the enrichment of bacteria harboring plasmid-borne ARGs relative to untreated systems, even though no antibiotics were detected in the latter. Metagenomic analysis has shown that genes involved in conjugative transfer and transposition are highly expressed in herbicide-exposed communities [6,50,51]. This implies that glyphosate pollution in water bodies could create “hotspots” for gene exchange among bacterioplankton, accelerating the dissemination of resistance traits across species and genera.

One specific pathway that is likely involved is the SOS response. SOS not only causes mutagenesis but also induces the excision of integrative conjugative elements and the replication of temperate phages that can carry ARGs. For instance, in a *Vibrio cholerae* model, SOS was triggered, and the transfer of the SXT integrative element (conferring multiple drug resistance) was greatly increased [52]. Although glyphosate was not tested in that study, it falls under the broader category of subinhibitory biocides that can activate HGT through stress responses. In summary, the consensus emerging is that glyphosate can amplify the spread of horizontal resistance by making bacterial communities more permissive to genetic exchange. This is especially concerning in environments where opportunistic pathogens mingle with environmental bacteria, as glyphosate could act as a silent matchmaker, encouraging the marriage of a resistance plasmid from an environmental strain with a pathogenic bacterium that then carries that resistance into the clinical setting.

### 2.6. Interactions with Metals and Co-Selection Dynamics

Glyphosate’s strong metal-chelating ability introduces another layer of complexity: it can bind micronutrient and heavy metal ions, thereby altering their availability to microorganisms [2]. This has two major implications for the AMR. First, heavy metals (such as Zn, Cu, and Cd) co-select for antibiotic resistance because many metal resistance genes are co-located with ARGs or share common regulatory systems [53]. If glyphosate use leads to the accumulation of metal ions in certain bioavailable forms, it could exacerbate metal stress on microbes, thus maintaining the selection pressure for metal- and antibiotic-resistant strains of microbes. Conversely, glyphosate may sequester metals and reduce their toxicity; for example, glyphosate can chelate Zn^2+^ required by metallo-β-lactamase (MBL) enzymes [54,55,56]. Intriguingly, a recent study showed that a glyphosate-based formulation inhibited the activity of MBL antibiotic-resistant enzymes in *P. aeruginosa* by chelating the Zn cofactor, similar to ethylenediaminetetraacetic acid (EDTA) [1]. The authors found that adding Roundup to MBL-producing strains restored their susceptibility to carbapenem antibiotics, highlighting a paradoxical effect: glyphosate can, in specific cases, reverse a resistance phenotype by depriving bacteria of essential metals [1]. Additionally, the same study noted that Roundup increased outer membrane permeability, enabling normally Gram-negative-impermeable antibiotics, such as vancomycin, to penetrate [1]. These contrasting outcomes highlight that glyphosate–metal interactions produce context-dependent effects that may promote or mitigate resistance-related traits. These findings further support the notion that glyphosate can exert direct biochemical effects on resistance-related mechanisms beyond its ecological role.

In agriculture, glyphosate is often used alongside copper-based fungicides or in soils with high iron or manganese concentrations. Glyphosate–metal complexes may persist in the soil and potentially affect microbial metal homeostasis [57]. Some soil bacteria possess plasmids that carry both metal resistance operons and ARGs [58]. If glyphosate causes these bacteria to experience metal starvation or overload, it could select for plasmid maintenance. Field surveys in China have linked long-term glyphosate application to higher ARG and MGE levels in soil, even when controlling for other factors [6]. It is plausible that part of this effect is due to co-selection; for example, glyphosate-tolerant bacteria might also need efficient metal efflux systems (which double as antibiotic efflux systems in many cases). Over time, this enriches the soil with multiple resistant species.

In summary, the chemical interactions of glyphosate with environmental factors (such as metals) create co-evolutionary pressures that can inadvertently enhance resistance. Glyphosate rarely acts alone, either by shared genetic mechanisms (metal and antibiotic efflux, plasmid linkages) or by altering the bioavailability of elements that bacteria need to grow (thus stressing them further). It is part of a cocktail of agrochemicals, and its legacy in ecosystems may be tied to combined effects, which is a frontier for understanding the influence of multiple pollutants on AMR. These findings highlight that glyphosate–metal interactions can lead to context-dependent outcomes, in which resistance-related phenotypes may be either enhanced or mitigated depending on environmental conditions, microbial physiology, and the specific resistance mechanisms involved. This bidirectional behavior underscores the need for caution when generalizing the effects of glyphosate on antimicrobial resistance.

### 2.7. Quorum-Sensing and Biofilm Effects

An emerging mechanistic dimension is the impact of glyphosate on quorum sensing (QS) and the formation of bacterial biofilms. Quorum sensing is a bacterial cell-to-cell communication mechanism via small signaling molecules (such as acyl-homoserine lactones in Gram-negative bacteria) that coordinate group behaviors, including biofilm development and the expression of virulence or conjugation genes [59]. Disruption of QS can lead to changes in the biofilm architecture and gene exchange dynamics of bacteria. Some studies suggest that bacteria modulate their QS signals in response to herbicides, possibly as an adaptive stress response. Nunes de Freitas et al. [60] showed that a *Pseudomonas* strain exposed to glyphosate and 2,4-D herbicide altered production of specific QS molecules. This is linked to the control of oxidative stress, as the strain may use QS-regulated mechanisms to mitigate ROS damage during herbicide exposure [60]. Such changes can influence biofilm formation; for instance, certain concentrations of glyphosate have been found to either inhibit or enhance biofilm formation in different bacterial species [41,61,62]. Biofilms are crucial in AMR evolution because they promote high cell density (favoring QS signaling) and facilitate HGT within protective matrices. If glyphosate encourages biofilm formation by environmental bacteria (perhaps by triggering mild stress that leads to communal aggregation), it could indirectly boost HGT and the persistence of ARGs in these biofilms. Conversely, if it inhibits biofilm formation or QS in certain beneficial bacteria, it may reduce their competitiveness over opportunistic bacteria.

One notable observation is that glyphosate-based formulations can increase membrane permeability to such an extent that even non-biofilm-forming planktonic cells become more accessible to glyphosate. Zerrouki et al. [1] demonstrated that the surfactant component of Roundup allowed vancomycin (a large molecule normally excluded from Gram-negative cells) to permeate, implying a general loosening of the outer membrane. Under these conditions, QS molecules may diffuse differently, potentially scrambling the usual communication gradients. Moreover, many conjugative plasmids encode QS-regulated systems that control their transfer frequencies [63,64]. Herbicide interference with QS could theoretically impede or accelerate plasmid transfers. Preliminary evidence suggests that *Enterobacter* adaption to herbicides may alter QS pathways to survive [65,66]. However, direct evidence linking glyphosate-induced QS alterations to AMR outcomes remains limited, and these mechanisms should be considered plausible but not yet fully validated pathways influencing resistance dynamics.

Another underexplored aspect is whether glyphosate residues in plant root exudates (for glyphosate applied to GMO crops) affect rhizosphere QS and biofilms of plant-associated microbes. The rhizosphere is a hotspot for horizontal gene exchange, and many plant pathogens form biofilms on roots. If glyphosate exudation disrupts normal root microbiome signaling, it could open niches for pathogenic bacteria that often carry ARGs [36]. For example, QS suppression could attenuate beneficial biofilms (such as biocontrol *Pseudomonas*), giving an edge to more herbicide-tolerant bacteria that might be less cooperative and more prone to gene acquisition.

In summary, although direct evidence of glyphosate affecting quorum sensing and consequent AMR outcomes is still limited, the possibility that glyphosate perturbs microbial communication is intriguing and warrants further investigation in the future. This could be linked to AMR by altering biofilm-mediated protection and the timing of conjugative transfer. Given that QS also controls the expression of efflux pumps and virulence factors [67], glyphosate interference could have a cascade of effects on how bacteria regulate stress and share genes within their communities. This area warrants more focused studies, as understanding QS disruption could reveal novel aspects of glyphosate’s impact on microbial ecology [60]. Collectively, these findings indicate that glyphosate and glyphosate-based formulations influence microbial adaptation through multiple interacting cellular responses, including metabolic stress, membrane remodeling, oxidative damage, and enhanced genetic exchange. These pathways are not isolated phenomena but form an interconnected network of evolutionary drivers that alter susceptibility patterns under realistic environmental conditions. These mechanistic interactions are summarized in a structured form in Table 1, highlighting the evidence base, microbial phenotypes, and antibiotic resistance-related outcomes reported in the literature. However, direct evidence linking glyphosate-induced QS alterations to AMR outcomes remains limited, and these mechanisms should be considered hypothesis-driven and require further validation.

## 3. Glyphosate Degradation, AMPA Ecology, and Potential Implications for Antimicrobial Resistance

The mechanisms described above illustrate how glyphosate exposure can directly modulate microbial physiology in ways that converge with the resistance-associated phenotypes of the target species. However, glyphosate toxicity represents only a small portion of its environmental footprint. A substantial fraction of glyphosate’s ecological legacy is mediated by its primary degradation product, aminomethylphosphonic acid (AMPA), which accumulates extensively in soils, sediments, and aquatic systems subjected to recurrent herbicide inputs [2]. In many agricultural and peri-urban environments, AMPA concentrations are equal to or exceed those of glyphosate, reflecting slower biodegradation rates, stronger adsorption to mineral surfaces, and reduced microbial turnover [66]. This persistence creates a distinct exposure regime characterized by chronic, low-intensity AMPA contamination rather than acute herbicide pulses, which may impose selective pressures that differ from those associated with parent compounds.

Glyphosate degradation is catalyzed by enzymes encoded by specific gene clusters, most notably the *phn* operon, which mediates carbon–phosphorus bond cleavage and phosphonate assimilation [68]. Bacteria harboring *phn* can access inorganic phosphate from glyphosate or AMPA, conferring a competitive advantage under phosphorus-limited conditions. Glyphosate oxidoreductase (GOX) converts glyphosate to AMPA and glyoxylate, integrating the degradation products into the central metabolism of the organism [2]. These catabolic capacities are phylogenetically uneven and are frequently associated with genera characterized by high genomic plasticity and environmental persistence, including *Pseudomonas*, *Burkholderia*, and *Acinetobacter* species [69,70]. Consequently, recurrent glyphosate inputs may selectively enrich taxa that combine phosphonate metabolism with traits linked to antimicrobial resilience, although this association remains largely inferred from ecological and genomic correlations rather than direct experimental validation of the resistance outcomes.

**Figure 1:**
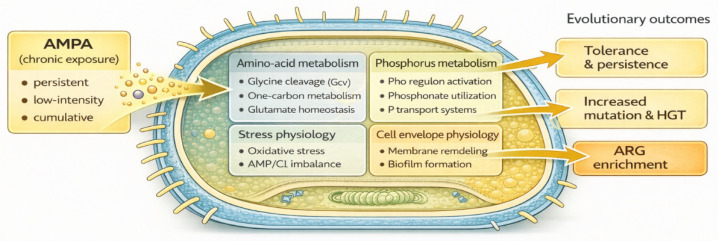
Conceptual model summarizing the putative mechanisms by which chronic aminomethylphosphonic acid (AMPA) exposure contributes to antimicrobial resistance in bacteria. AMPA accumulation interferes with one-carbon metabolism and glycine cleavage pathways, activates the Pho regulon and phosphonate utilization systems, and triggers oxidative and stringent stress responses in microbial cells. These metabolic and regulatory shifts may contribute to envelope remodeling, biofilm formation, mutagenesis, and horizontal gene transfer, thereby shaping the long-term AMR trajectory. Abbreviations: Pho, phosphate regulon; Gcv, glycine cleavage system; AMPA, aminomethylphosphonic acid; ARGs, antibiotic resistance genes; HGT, horizontal gene transfer. This figure was developed with the assistance of the Gemini 4 platform (Google DeepMind) and was subsequently refined and validated by the author.

Beyond specific catabolic pathways, phosphonate exposure intersects with global regulatory systems that govern nutrient stress, particularly the Pho regulon. Activation of pho under phosphate limitation induces not only phosphonate transport and metabolism genes but also pathways involved in membrane remodeling, efflux modulation, and biofilm formation [71,72]. These responses are energetically costly and are typically associated with a broad-spectrum stress tolerance. If AMPA accumulation constrains bioavailable phosphorus pools, repeated activation of pho-mediated adaptive states may indirectly select for microbial strategies that overlap with resistance-associated phenotypes, even in the absence of antibiotic pressure (Figure 1). However, direct empirical evidence linking Pho activation under AMPA exposure to antimicrobial resistance outcomes remains limited, and these mechanisms should be interpreted as plausible, but not yet fully validated pathways.

AMPA may further reinforce these selective dynamics through metabolic interference. In plants, AMPA inhibits glycine decarboxylase (GDC), leading to glycine accumulation and glutamate depletion, with downstream effects on chlorophyll biosynthesis and energy metabolism [73]. Although bacteria do not synthesize chlorophyll, glycine cleavage systems (Gcv) play an analogous central role in one-carbon metabolism, redox balance, and amino acid homeostasis [74]. Perturbation of glycine flux or Gcv activity by AMPA disrupts the intracellular glycine–glutamate balance, indirectly affecting nitrogen assimilation, central carbon metabolism, and oxidative stress regulation (Figure 1). These metabolic bottlenecks activate stringent response pathways and promote slow growth or persistent states, which are strongly associated with tolerance to antibiotics. Consistent with this framework, glyphosate exposure induces persistent phenotypes without increasing the minimum inhibitory concentrations [15]. Nevertheless, direct evidence for AMPA-induced metabolic disruption leading to resistance-associated phenotypes in bacteria remains scarce and is largely extrapolated from related systems.

Metal chelation provides an additional axis linking AMPA metabolism to the evolutionary dynamics. AMPA binds to divalent cations that are essential for enzymatic activity and redox homeostasis, potentially inducing oxidative stress and destabilizing metal-dependent metabolic processes [2]. Oxidative stress activates the SOS response, elevating mutation rates and mobilizing genetic elements. Human biomonitoring studies have associated AMPA exposure with oxidative DNA damage [75], supporting the plausibility of comparable effects in gut microbial systems in humans. If AMPA generates a background of chronic oxidative or nutritional stress, it may accelerate adaptive evolution by increasing the mutational supply and genetic turnover, indirectly favoring resistance emergence. However, extrapolation from host-associated or eukaryotic systems to environmental microbiomes requires caution and further research.

In summary, the ecological consequences of glyphosate degradation extend beyond direct toxicity to the evolutionarily filtering of microbial communities. Bacteria capable of phosphonate metabolism may be favored in AMPA-rich environments because they exploit alternative nutrient pools, tolerate metabolic stress, and operate regulatory architectures that support survival under fluctuating conditions. Many of these traits are characteristic of opportunistic and multidrug-resistant lineages, suggesting that AMPA exposure may reshape community composition toward taxa with pre-adaptations relevant to antimicrobial resistance. Importantly, these associations do not demonstrate direct selection for antimicrobial resistance but rather indicate overlapping adaptive strategies that may co-occur with resistance.

Finally, the genomic architecture of phosphonate metabolism raises important concerns regarding co-selection of phosphonate-degrading bacteria. The *phn* operon is frequently located on mobile genetic elements, including plasmids and genomic islands, particularly in bacteria that inhabit dynamic and polluted ecosystems. Therefore, the horizontal transfer of phosphonate metabolism genes may disseminate genomic frameworks that facilitate ARG acquisition or maintenance, even in the absence of direct physical linkages. Although evidence for the systematic co-localization of *phn* clusters and ARGs remains limited, the alignment of ecological incentives (survival in phosphonate-rich environments and tolerance to multiple stressors) creates a selective landscape in which co-association may be plausible. Collectively, these observations support a conceptual shift in our understanding of pesticide-driven AMR. AMPA is unlikely to act as an antibiotic-like selective agent; rather, it may function as a slow, pervasive ecological filter operating through metabolic stress, nutrient limitation, redox imbalance, and genetic mobility. Because AMPA is persistent, chemically active, and globally distributed, its role as a potential long-term driver of resistome reorganization warrants targeted empirical investigation, particularly through metagenomic, transcriptomic, and functional assays that can link metabolic stress pathways to resistance dynamics [1].

## 4. Impacts on Environmental Microbiota and Resistomes

### 4.1. Soil and Rhizosphere Microbiomes

Agricultural soils are the primary recipients of glyphosate, with many fields experiencing repeated applications over the years. These diffuse exposures shape the soil microbiome and its reservoir of resistance genes. Reported concentrations in cultivated soils commonly range from tens to thousands of μg/kg, with maxima around 2 mg/kg in some agricultural systems; AMPA often occurs at similar or even higher levels [4,5,76]. Recent field and microcosm studies have indicated that glyphosate application is associated with the enrichment of soil resistomes without obvious signs of ecological disruption. Liao [6] found an increased abundance of ARGs (spanning multiple drug classes) and MGEs in herbicide-treated soils, despite little change in the broad bacterial community composition. Glyphosate had the strongest effect among the herbicides tested (glufosinate and dicamba were also studied), which correlated with its intensive use in these soils [6]. Soil samples with a history of glyphosate use across diverse Chinese provinces consistently showed higher ARG/MGE levels than soils with no herbicide history, reinforcing that this phenomenon is not restricted to a single location or soil type.

Mechanistically, soil is a complex matrix in which glyphosate can adsorb minerals, biodegrade, or remain available to microbes in the pore water. Glyphosate-tolerant soil bacteria (including some *Pseudomonas*, *Acinetobacter*, and Gram-positive rods) likely proliferate after glyphosate application [77], and some of these are known to harbor intrinsic or acquired antibiotic resistance. For example, *Pseudomonas* spp. are naturally resilient and often carry efflux pumps that confer antibiotic tolerance [78]. Consequently, shifts in microbial composition may indirectly increase ARG abundance via ecological selection. Simultaneously, these ecological dynamics may interact with the cellular-level mechanisms described in Section 2, reinforcing resistance-associated phenotypes in affected communities.

In agricultural soil studies, glyphosate treatments have been associated with a decline in beneficial *Bacillus* and *Nitrobacter* populations and an increase in opportunistic species such as *Sphingomonas* and *Burkholderia* [61]. Notably, some *Burkholderia* species are both herbicide degraders and multidrug-resistant opportunistic pathogens. Thus, even modest compositional shifts may alter the functional profiles of soil microbiomes, particularly with respect to stress tolerance and resistance potential. However, these responses are highly dependent on soil physicochemical properties, exposure history, and co-occurring environmental stressors, limiting their generalization across systems.

Glyphosate can have direct and indirect effects in the rhizosphere (a narrow zone around plant roots teeming with microbes). Glyphosate is sometimes exuded by the roots of treated plants or released upon the decomposition of sprayed weeds [2]. Rhizosphere microbiota play roles in nutrient cycling and plant defense, and glyphosate-induced dysbiosis may affect plant health and ARG propagation. Studies have reported the suppression of symbiotic nitrogen-fixing bacteria (e.g., *Rhizobium* spp.) and mycorrhizal fungi, alongside the enrichment of bacteria capable of using glyphosate as a nutrient [8,79,80]. These shifts may indirectly influence resistance dynamics by altering competitive interactions and ecological niches of the host. Moreover, if mutualists are reduced, plant stress may invite more disease-causing bacteria, which often carry ARGs (e.g., phytopathogenic *Pseudomonas* or *Xanthomonas*, which often harbor efflux-mediated antibiotic resistance). Furthermore, plasmids carrying both plant-interactive traits and ARGs could spread in the rhizosphere when changes in community composition occur. Some studies have detected an increased prevalence of class 1 integrons (common carriers of ARG cassettes) in the rhizosphere of glyphosate-tolerant GMO crops compared to their non-GMO equivalents [6,81], indicating a link between glyphosate usage and ARG carriage in root-associated bacteria in the rhizosphere.

It is important to note that soil conditions (pH, clay content, and organic matter) modulate glyphosate bioavailability and its microbial effects on soil. Glyphosate tightly binds to clays and iron/aluminum oxides; therefore, microbes in such soils may experience lower effective doses [82]. This could explain why some studies have found minimal changes in soil microbiomes after glyphosate application [83,84]. However, repeated application can saturate the binding sites or create microenvironments (e.g., decaying weed residues) with high levels of free glyphosate. Microbial communities can be drastically altered in these niches. From a One Health perspective, soil is a critical compartment because it can disseminate ARGs via dust, water runoff, and food crops. Accordingly, the influence of glyphosate on soil resistomes should be interpreted as part of a broader network of interacting environmental and mechanistic drivers, rather than an isolated causal factor.

### 4.2. Aquatic Systems: Freshwater, Wastewater, and Sediments

Glyphosate and its formulations are washed into rivers, lakes, and wetlands via agricultural runoff and drainage systems. In water bodies, glyphosate has a half-life on the order of weeks and can reach concentrations from ~0.01 to 58 μg/L under monitored environmental conditions, whereas runoff studies have reported concentrations exceeding 1000 μg/L in highly impacted systems [10,11]. Microbial communities in these aquatic systems have shown responses to glyphosate that mirror some soil trends, such as the enrichment of certain proteobacterial groups and their associated ARGs.

Barbosa da Costa [49] conducted controlled freshwater microcosm experiments in which glyphosate-based herbicide (at environmentally relevant concentration) was added to natural lake water communities. After repeated exposure, bacterioplankton communities developed higher relative abundances of multiple ARGs (particularly sulfonamide, β-lactam, and tetracycline resistance genes) than unexposed controls. Interestingly, this enrichment occurred without significant shifts in overall microbial diversity, suggesting that resistance dynamics may arise from shifts in relative abundance or HGT rather than large-scale community restructuring. Metagenomic analysis has indicated an increase in plasmids and transposase-associated genes under herbicide exposure [6,49,85], supporting a role for gene mobility. However, much of this evidence is derived from controlled experimental systems, and the extent to which these patterns occur under natural environmental variability remains uncertain. Factors such as hydrological dynamics, nutrient availability, and the presence of co-contaminants can strongly influence microbial responses.

Glyphosate may also play a role in wastewater treatment plants (WWTPs), where it co-occurs with a wide range of chemical stressors [1,12]. AMPA is frequently detected in WWTP influents and effluents and can accumulate in activated sludge [1]. Sludge bacteria capable of phosphonate metabolism may be selectively favored, and these taxa (e.g., *Acinetobacter* and *Pseudomonas*) are also associated with multidrug resistance. Nevertheless, the specific contribution of glyphosate relative to other co-occurring stressors in WWTPs remains difficult to isolate, given the complexity of these systems.

**Figure 2:**
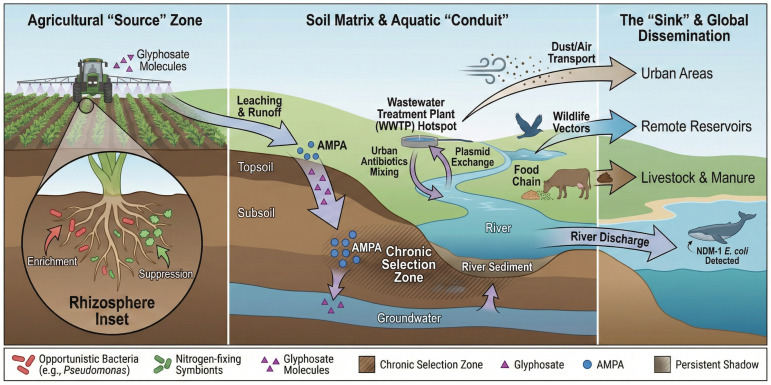
Glyphosate-driven antimicrobial resistance dynamics in aquatic systems. Glyphosate and its metabolite AMPA impose distinct selective pressures on freshwater, wastewater treatment systems, and aquatic sediments. Through hydrological connectivity, resuspension, and biological transport, resistance determinants selected in one compartment may propagate across aquatic networks, highlighting sediments and treatment systems as critical reservoirs and amplification nodes within the One Health Framework. This figure was developed with the assistance of the Gemini 4 platform (Google DeepMind) and subsequently refined and validated by the author.

In aquatic sediments, glyphosate tends to strongly adsorb onto particulate matter and persists over extended timescales, reflecting its high affinity for mineral surfaces and organic particles. Consequently, sediments can function as long-term reservoirs for glyphosate and its metabolite AMPA, as well as antimicrobial resistance determinants, including ARGs and mobile genetic elements [4,11,82]. Sediment-associated bacteria, frequently embedded in particle-attached biofilms, are therefore exposed to sustained contact with herbicide residues under conditions that differ markedly from those in the overlying water column (Figure 2). These environments often host dense biofilms under reduced oxygen conditions, potentially favoring the persistence of resistance determinants. Although field studies have consistently documented the co-occurrence of herbicides and ARGs in sediments [4], direct causal relationships between glyphosate accumulation and ARG enrichment remain unresolved. Sediments represent critical zones where prolonged chemical exposure, reduced redox conditions, and high microbial densities may favor the stabilization and persistence of resistance determinants. Importantly, sediment-associated ARGs are not necessarily confined to the benthic compartments. Physical disturbances, such as storm events, flooding, and dredging, can resuspend contaminated particles, facilitating the transfer of resistance genes back into the water column [86]. In parallel, benthic invertebrates may act as biological vectors, incorporating sediment-associated microbiota and resistance elements into the aquatic food webs. Together, these findings suggest that sediments may function as dynamic reservoirs and secondary sources of antimicrobial resistance, underscoring the need to explicitly consider sediment–water coupling in assessments of glyphosate-driven resistance risks.

Overall, aquatic ecosystems demonstrate that glyphosate-associated resistance dynamics are influenced by hydrological connectivity and multi-compartment interactions within these ecosystems. Water systems act as conduits linking terrestrial, aquatic, and marine environments and facilitate the dissemination of resistance traits. While experimental evidence supports the association between herbicide exposure and resistome shifts, causal relationships in natural systems remain context-dependent and are influenced by interacting environmental factors.

### 4.3. Non-Target Effects on Wildlife and Food Chains

As glyphosate-based herbicides pervade the environment, non-target organisms, from microbes to mammals, are exposed to glyphosate exposure. This has ripple effects on the microbial ecology within these hosts and at the interfaces between the environment and animals and humans. For example, earthworms and deer exhibit gut microbiome changes linked to glyphosate residues [23,24]. These changes not only affect host physiology and digestion but may also alter susceptibility to opportunistic infections through the disruption of the microbiome. Wild birds that forage in treated fields or contaminated water can acquire resistant bacteria from these environments and spread them over long distances. Gomes et al. [84] reported a multidrug-resistant *E. coli* (CTX-M β-lactamase-producing ST602) in wild migratory birds in South America, which carried a “wide resistome” including genes likely derived from anthropogenic sources. Birds can acquire such strains through contact with soil or water containing manure, antibiotics, and possibly herbicide-stressed bacteria. The significance here is that wildlife can bridge ecosystems: a bird feeding in a glyphosate-treated farm pond during the day might roost in a city at night, thereby depositing resistant bacteria via its feces. If glyphosate exposure contributes to the enrichment or persistence of such bacteria in environmental reservoirs, it may indirectly influence the pool of migrating resistant bacteria.

Similarly, livestock and companion animals can be affected by this disease as well. Glyphosate residues are common in feed (e.g., glyphosate-tolerant GM corn/soy feed can contain ppm-level residues) [87]. In pigs, studies have shown varying outcomes: one investigation found subtle metabolic effects but no significant changes in community taxonomy or short-chain fatty acids when exposed to Roundup formulations [88], while another study reported that weaned piglets did not develop gut dysbiosis when fed glyphosate-amended diets at maximum residue levels [89]. However, other studies have demonstrated clear microbiota alterations: glyphosate exposure in rats significantly decreased *Firmicutes* and *Lactobacillus* while enriching potentially pathogenic bacteria [86], and Roundup formulations altered bacterial and fungal populations in rat cecum microbiota [90]. In broiler hens, chronic glyphosate exposure temporarily decreased cecal propionate concentrations and durably affected the cecal microbiome by inhibiting *Barnesiella* and favoring *Alloprevotella* [87]. Overall, beneficial intestinal bacteria are often negatively affected, whereas pathogenic species may be enhanced [8]. Such shifts may influence the microbial community structure and create ecological conditions that favor the persistence or exchange of resistance determinants. If pathogenic bacteria such as *Salmonella* or *E. coli* O157 survive better or exchange more plasmids in an herbicide-perturbed gut, the risk of foodborne illness with resistant strains increases. Indeed, one hypothesis for the high incidence of certain resistant infections in regions with relatively low antibiotic usage (such as parts of the tropics) is environmental drivers, such as pesticides [3]. For example, Brazil has both heavy pesticide use and rising AMR in clinical isolates. Although correlation does not imply causation, it warrants an examination of whether farmworkers or rural populations have a higher carriage of resistant gut bacteria associated with herbicide exposure than urban populations.

Another channel is through fish and other aquatic organisms. Glyphosate runoff can affect microbial communities in fish farms or natural aquatic habitats, potentially altering the pathogen landscape that fish are exposed to [88]. If herbicide stress selects for antibiotic-resistant *Aeromonas* or *Vibrio* in water, fish could become colonized/infected with these bacteria, and these fish (or their waste) would propagate this cycle. Additionally, humans who consume such fish or use these waters for recreation may be at risk. The One Health implications are clear: the ecological impacts of glyphosate cannot be isolated from human health. A shift in the soil or water microbiome can lead to different exposures of humans to resistant bacteria, whether through food, water, or contact with animals. The discovery of environmental superbugs, such as NDM-1 *E. coli* in a whale or drug-resistant *Pseudomonas* in remote wetlands, should serve as warning signs that resistant bacteria do not respect geographical boundaries [14]. Glyphosate, although not acting as the sole driver, may function as one of several interacting environmental pressures that shape these dynamics. Table 2 provides a synthesis of these ecological outcomes, documenting community-level responses, resistome changes and exposure scenarios across distinct environmental matrices.

## 5. One Health Perspectives and Implications

The evidence reviewed above paints a concerning picture in which glyphosate and its formulations serve as a pervasive environmental pressure with the potential to foster antimicrobial resistance across the ecosystem–animal–human interface. Glyphosate should not be interpreted as an isolated driver of AMR, but rather as a component of complex, multi-stressor systems that influence microbial evolution across environmental compartments. Under the One Health framework, environmental health is intrinsically linked to both animal and human health. Glyphosate’s contribution to AMR exemplifies this connection:

- In the environment (ecosystem health): Glyphosate-enriched resistomes in soil and water act as ARG reservoirs. These genes can be acquired by opportunistic environmental bacteria, some of which can cause infections in humans or animals (either directly, as with *Pseudomonas* and *Aeromonas*, or indirectly as sources of resistance genes for true pathogens). The environment also serves as a mixing bowl where genes from clinical sources (e.g., via wastewater) meet agricultural and wild-type bacteria, with glyphosate possibly catalyzing this exchange. The result can be novel resistance gene combinations that eventually find their way back to clinics (e.g., via contaminated produce or water). In essence, the environmental misuse of chemicals parallels the misuse of antibiotics, driving the development of resistance.
- In animals (animal health): Animals, including livestock, companion animals, and wildlife, often share both the environment and close contact with humans. If their microbiomes are skewed by glyphosate (or if they acquire resistant bacteria from the environment), these animals can become asymptomatic carriers of AMR or suffer from infections that are difficult to treat. For example, a farm animal grazing on glyphosate-treated pastures could ingest herbicide-stressed soil microbes that carry ARGs and later shed them through manure. Manure can be spread on fields or infect farmworkers. Wild birds, as highlighted, can disseminate ARGs globally; a bird does not need antibiotics to pick up a resistant bug if glyphosate and other factors have already selected it in the environment. Therefore, protecting animal health requires recognition of herbicide exposure as a possible risk factor for AMR proliferation in herds and in wildlife populations.
- In humans (public health): humans ultimately encounter these resistant strains through various pathways: contaminated food (vegetables grown in ARG-rich soil or meat from animals with altered gut flora), water (drinking or recreation in water downstream of agricultural areas), and direct contact (farmers and gardeners have repeated skin and inhalation exposure to soil and dust that may contain herbicide-selected microbes). In particular, rural agricultural communities may be at triple jeopardy: they handle glyphosate, live in an environment where it accumulates, and often lack advanced sanitation, making them more likely to exchange microbes with their environments. The One Health view stresses that interventions to curb AMR must extend beyond hospitals to the fields and rivers. For glyphosate, this could mean re-evaluating its usage guidelines, considering spray practices that minimize runoff, and developing formulations that are less likely to induce stress responses in bacteria.

Importantly, One Health refers to interdisciplinary surveillance. Currently, AMR monitoring is robust in clinical settings but sparse in environmental settings. The findings of Liao, H. et al. [6], Barbosa da Costa et al. [49], and others suggest that tracking glyphosate residues alongside ARG prevalence in various matrices can serve as an early warning system. For instance, if a region shows an increase in glyphosate levels in water and concurrently an increase in ARGs in wildlife or farm animals, targeted actions (such as mitigating runoff or adjusting herbicide application timing) may prevent these ARGs from reaching hospitals. This has regulatory implications for industries. Traditionally, pesticide risk assessments have focused on toxicity to non-target organisms (frogs, bees, etc.) and ignored the evolutionary pressure on microorganisms. Our review underscores the relevance of AMR evolution as an endpoint in chemical risk assessment, within the One Health framework. Just as regulators consider how a pesticide might select for resistant pests or weeds, they should consider the selection of resistant microbes. This could involve studies on whether long-term sublethal exposure to a new pesticide affects the resistome or conjugation rates of microbial communities. Some experts have begun calling for such integration, arguing that environmental concentrations previously deemed “safe” (for acute toxicity) may still have ecosystem-level consequences by accelerating AMR [8].

Another One Health angle is potential mitigation: could we leverage any positive effects of glyphosate (such as its chelation of metals, like inactivating MBLs) in a controlled manner to combat AMR? The synergism observed by Zerrouki et al. [1], in which glyphosate made *P. aeruginosa* more susceptible to colistin and carbapenems, suggests that glyphosate or its analogs could be repurposed as antibiotic adjuvants in specific scenarios. However, this would require extreme caution; using an herbicide in patients is not practical, but understanding this mechanism (metal chelation increasing permeability) could inspire new treatments for cystinosis. This also serves as a reminder that the interactions between pollutants and pathogens are inherently complex. Glyphosate may simultaneously drive resistance in some contexts while suppressing it in others. A One Health approach must navigate these nuances, aiming to maximize co-benefits (if any) and minimize the risks. In conclusion, the association between glyphosate and AMR exemplifies a One Health challenge in which agricultural practices affect microbial evolution and potentially clinical outcomes. This urges a more holistic stewardship of antimicrobials and antimicrobials-by-proxy (such as biocides and pesticides). To protect the efficacy of antibiotics, society may need to broaden the scope of “antibiotic misuse” to include the heavy use of compounds such as glyphosate that, while not antibiotics themselves in the traditional sense, can undermine antibiotic effectiveness by sculpting the resistome in our shared environment.

## 6. Conclusions and Future Directions

The body of evidence amassed from 2019 onward provides compelling support that glyphosate, its metabolites such as AMPA, and commercial formulations (for example, Roundup) are non-antibiotic drivers of the evolution of antimicrobial resistance. Through diverse mechanisms, including metabolic inhibition, stress response induction, and enhanced HGT, glyphosate exposure may select for antibiotic-resistant bacteria and genes in the environment. These effects have been documented in soil microbiomes, freshwater communities, animal guts, and model laboratory strains, lending credibility to the concept that even ostensibly unrelated chemicals can contribute to the global AMR crisis. Importantly, the review highlights that AMR should be viewed not just as a clinical or pharmaceutical issue, but as an ecological and evolutionary one, where pollution and human activities play a significant role in the selection of resistance mechanisms.

However, it is important to explicitly acknowledge the current limitations of this field. Much of the available mechanistic evidence derives from controlled laboratory experiments or mesocosm systems using model organisms, which may not fully represent the complexity and variability of the natural environment. In real-world settings, microbial responses are shaped by interacting factors such as physicochemical conditions, community composition, hydrological dynamics, and co-occurring stressors, all of which can modulate both exposure and biological outcomes of pollutants. Furthermore, direct in vivo and field-based validations linking glyphosate exposure to antimicrobial resistance dynamics are limited. These constraints highlight the need for integrative approaches that combine controlled mechanistic studies with long-term environmental monitoring and multi-omics analyses to better understand the ecological relevance of these processes. Future research directions emerging from our critical analysis include the following.

- Elucidating Underexplored Mechanisms: Some hypothesized pathways, such as glyphosate-induced quorum-sensing disruption or direct oxidative DNA damage, require further experimental verification. Do QS-regulated conjugative plasmids transfer more readily under glyphosate stress? Does glyphosate increase the mutation rate via specific oxidative lesions or replication errors? Advanced omics and single-cell analyses may provide insights into these issues. Furthermore, the intriguing finding that glyphosate induces bacterial persistence (tolerance) but not heritable resistance raises questions regarding the extent of this phenomenon across species and its long-term implications. For example, could recurrent glyphosate exposure in soil create a seedbed of “persisted” cells that survive both herbicides and antibiotics, thereby increasing the likelihood of subsequent resistance mutations?
- Assessing AMPA and Formulants: The main analog and breakdown product, AMPA, is ubiquitous in glyphosate-treated environments; however, its biological effects have not yet been well characterized. Does AMPA exert a similar selective pressure on microbes, or is it benign? Its strong metal-chelating properties could influence microbial communities in a manner similar to that of glyphosate. Additionally, surfactants and co-formulants in commercial herbicides (such as POEA in Roundup) may have independent or synergistic effects on bacteria. Some studies suggest that formulants can be more toxic to microbes than glyphosate alone (perhaps explain why certain combinations had stronger effects on *P. aeruginosa*). Determining the contributions of active ingredients and formulants is critical for regulatory decision making. For instance, glyphosate can be formulated to minimize its impact on microbial communities in the soil.
- Long-Term and Low-Dose Exposure Studies: Most laboratory experiments use acute exposure from day to week. Environmental microbes have been exposed to low-dose chronic exposure for several years. Long-term microcosm or field studies (such as multi-year experiments varying glyphosate application frequency) are needed to observe cumulative and possibly subtle shifts in the resistomes. Such studies should also incorporate recovery periods to determine whether the effects persist or whether reversible dysbiosis occurs when glyphosate is removed from the environment. The durability of glyphosate-driven resistance (do ARG abundances fall if a field switches to organic practices for a few years?) This is a highly practical question regarding land-use management.
- Integration with Monitoring Programs: Pesticide residue surveillance should be coupled with resistome analysis. Modern metagenomic sequencing allows the tracking of dozens of ARG markers in a sample. Regulators could include ARG screening in the environmental impact assessments of herbicide use. An index of “resistance pressure” can be envisioned for a given site that incorporates pesticide and antibiotic usage and observed ARG proliferation. If certain ARGs (e.g., vancomycin or carbapenem resistance genes) start to increase in environments with heavy glyphosate use, this should trigger further investigation.
- Mitigation Strategies: If glyphosate is confirmed as a significant contributor to AMR, several mitigation strategies can be implemented. One approach is to develop bioremediation strategies, such as promoting the growth of glyphosate-degrading bacteria in runoff zones, to break down herbicides before they affect the broader microbial communities. However, it is important to ensure that the degraders do not carry ARGs away. Another strategy is crop and soil management, such as alternating glyphosate with other weed control methods to avoid constant pressure or using buffer strips to intercept herbicide runoff (protect water microbiomes). Additionally, the exploration of herbicides and growth regulators that induce lower AMR may be necessary.
- Cross-Disciplinary Policy: This review underscores the need for cross-disciplinary collaboration between agronomists, microbiologists, ecotoxicologists, and clinicians. Policies aimed at combating AMR (such as national AMR action plans) should extend their scope to include environmental drivers, such as pesticides. Conversely, pesticide regulatory frameworks (usually separate from health sectors) should integrate knowledge from the medical and microbiological domains. A concrete example is the requirement to assess whether a new herbicide could enhance HGT among bacteria, similar to the assessment of carcinogenicity or endocrine disruption.

The panel in Table 3 outlines the key research priorities, ranging from the role of AMPA and surfactants to the reversibility of resistome shifts and the integration of evolutionary endpoints into regulatory frameworks. In conclusion, the relationship between glyphosate and antimicrobial resistance is a cautionary tale of unintended consequences that must be addressed in future studies. A molecule designed to target plants has been shown to affect the microbial world in ways that challenge the use of antibiotics under specific conditions. Recognizing this link also offers an opportunity to broaden our perspective on AMR as a planetary health issue that requires the stewardship of all antimicrobials, whether used in medicine or the field. By pursuing the research directions outlined and translating the findings into practice (e.g., improved guidelines for herbicide use and integrated pest and pathogen management strategies), we can mitigate the collateral damage of glyphosate on the resistome while still addressing the agricultural needs of society. Ultimately, safeguarding antibiotic efficacy will require such holistic approaches, befitting the One Health principle that the health of humans, animals, and ecosystems is deeply interdependent.

## Figures and Tables

**Table 1:** Mechanistic pathways linking glyphosate exposure to antimicrobial resistance.

Mechanism	Evidence Type	Microbial Effect	Antibiotic Resistance-Related Outcomes	Evidence Strength	Representative Studies
EPSPS inhibition → stringent response	In vitro; transcriptomics	Growth arrest; persistence	Tolerance without MIC increase	Experimental (direct)	[14]
Multidrug efflux pump induction	In vitro; RNA-seq	Increased efflux; reduced permeability	Increased MIC; cross-resistance	Experimental (direct)	[28,29]
Membrane remodeling	In vitro	Reduced porins; altered fatty acid composition	Reduced antibiotic uptake	Experimental (direct)	[6]
Oxidative stress and SOS response	In vitro; biochemical assays	DNA damage; hypermutation	Increased mutation frequency; resistance emergence	Experimental (direct)	[40]
Increased horizontal gene transfer	Mesocosm; soil; freshwater; laboratory mating assays	Enhanced conjugation; plasmid transfer; gene mobility	Dissemination of ARGs	Experimental (lab + mesocosm)	[6,42,48]
Microbial dysbiosis	Soil; gut; aquatic systems	Shift toward tolerant or opportunistic taxa	ARG enrichment; resistome restructuring	Observational/ecological	[6,17,22]
Metal chelation	Chemical; microbiological	Altered metal availability; enzyme inhibition	Co-selection or reversal of resistance phenotypes	Mechanistic (context-dependent)	[1]
Quorum sensing and biofilm modulation	In vitro	Altered communication; biofilm formation	Potential persistence and gene transfer enhancement	Limited/hypothesis-supported	[34]

**Table 2:** Ecological impacts of glyphosate exposure on microbiomes and resistomes in different environments.

Environment	Effect on Microbial Community	Effect on ARGs/MGEs	Exposure Conditions	Representative Studies
Agricultural soil	Selective enrichment	↑ ARGs, integrons, plasmids	Long-term	[6]
Rhizosphere	Shift to degraders	↑ ARG carriers	Root exudates + herbicide	[34]
Freshwater	Stable α-diversity	↑ plasmid mobility	Mesocosm	[48]
Sediments	AMPA accumulation	↑ integrons	Watersheds	[83]
Wildlife gut	Dysbiosis	↑ ARG load	Dietary	[22]
Earthworm gut	Altered core taxa	↑ determinants	Soil exposure	[23]
Livestock gut	Altered microbiota	Potential ARG enrichment	Feed contaminants	[90]

Arrows indicate the direction of the changes reported in the literature (↑ increase). ARGs, antibiotic resistance genes; MGEs, mobile genetic elements. The reported effects are based on representative studies and may vary depending on the exposure conditions and environmental context.

**Table 3:** Key knowledge gaps and emerging research priorities for understanding glyphosate-associated antimicrobial resistance across environmental, animal, and human systems (One Health perspective).

Research Gap	Rationale	Recommended Approaches	Expected Impact
AMPA-specific toxicity	Unknown	Comparative genomics	High
Surfactant effects	Formulation dependent	Fractionated assays	High
Long-term low-dose	Realistic exposure	Field trials	High
Co-exposures	Common in agriculture	Factorial designs	Very high
QS and HGT link	Understudied	Molecular assays	Medium
Network rewiring	Ecological	Time-series metagenomics	High
Reversibility	Unknown	Withdrawal experiments	High
Wildlife vectors	Limited data	Monitoring	Medium
Regulatory frameworks	No evolutionary endpoints	AMR endpoints in ERA	Very high

## Data Availability

No new data were created or analyzed in this study.
